# Global burdens of diabetes mellitus in adolescents from 1990 to 2023 and future trend predictions: An analysis of the Global Burden of Disease study 2023

**DOI:** 10.1371/journal.pone.0358475

**Published:** 2026-09-16

**Authors:** Xinyi Qiu, Limei Guan, Hui Liu

**Affiliations:** College of Clinical Medicine for Obstetrics & Gynecology and Pediatrics, Fujian Medical University, Fujian Children’s Hospital (Fujian Branch of Shanghai Children’s Medical Center), Fuzhou, Fujian, China; Lorestan University, IRAN, ISLAMIC REPUBLIC OF

## Abstract

**Background:**

Adolescent diabetes mellitus (DM) constitutes a significant global public health challenge. This study seeks to quantify the burden of DM in adolescents aged 10–19 years from 1990 to 2023 and to forecast trends in incidence, prevalence, mortality, and disability-adjusted life years (DALYs) through 2040.

**Methods:**

Epidemiological data for DM among adolescents in 204 countries and territories were obtained from the Global Burden of Disease (GBD) 2023 database. The global burden was evaluated using age-standardized incidence rate (ASIR), age-standardized prevalence rate (ASPR), age-standardized mortality rate (ASMR), and age-standardized DALY rate (ASDR), stratified by region, sex, age, and Socio-demographic Index (SDI). Health inequality was assessed using the slope index of inequality (SII) and concentration index (CI). Future trends were forecast using an autoregressive integrated moving average (ARIMA) model with model-specific validation (AICc, Ljung-Box test).

**Results:**

From 1990 to 2023, global adolescent DM burden increased substantially. ASIR, ASPR, and ASDR rose consistently, whereas ASMR remained relatively stable with a slight decline. In 2023, an estimated 6.07 million adolescents were living with DM (ASPR: 458.07 per 100,000). The highest ASPR was observed in low-SDI regions (482.25 per 100,000), followed by high-SDI regions (442.81 per 100,000). Low-SDI regions also carried the highest DALY and mortality burdens. Males progressively exceeded females in both incidence and prevalence by 2023, although this observed sex reversal may partly reflect changes in screening and diagnostic practices. The overall burden increase was primarily driven by adolescents aged 15–19 years. ARIMA forecasts indicate that ASIR, ASPR, and ASDR are projected to continue increasing through 2040, while ASMR is expected to remain low and stable.

**Conclusions:**

The burden of adolescent DM increased considerably from 1990 to 2023, with marked heterogeneity by age, sex, and geography. The rising non-fatal burden (incidence, prevalence, DALYs) contrasts with stable mortality, suggesting improvements in acute care alongside growing morbidity. Persistent health disparities and projected increases underscore the need for age-, sex-, and region-specific public health strategies.

## Introduction

Diabetes mellitus (DM) is among the most common and swiftly increasing disorders globally [1,2]. Extended duration of diabetes mellitus can result in dysfunction and failure of various organs, particularly the eyes, kidneys, nerves, heart, and blood vessels. The latest data from the International Diabetes Federation (IDF) Diabetes Atlas (11th Edition, 2025) estimates that around 589 million adults aged 20–79 globally were living with diabetes in 2024, representing 11.1% of the global population in this age group, with nearly half undiagnosed. The frequency of diabetes mellitus escalates with age, peaking at 24.8% among adults aged 75–79 [3,4]. The incidence of diabetes mellitus is anticipated to reach 853 million by 2050, mostly due to aging demographics, increasing obesity prevalence, and lifestyle influences. Significantly, low- and middle-income nations are projected to bear 75% of this illness burden.

Type 1 diabetes mellitus (T1DM) is the most common form of diabetes mellitus among adolescents, with its highest frequency occurring during this developmental stage and a mean age of diagnosis approximately around 12 years. Simultaneously, escalating rates of childhood obesity have resulted in a heightened incidence of type 2 diabetes mellitus (T2DM) among adolescents across multiple nations. T2DM is estimated to account for approximately 32% of DM cases within this demographic. Increasing researches indicate that early-onset DM correlates with a heightened risk of premature complications, significantly impacting patients’ quality of life and long-term health outcomes [5,6]. Research conducted by the Collaborative Group on Emerging Risk Factors indicates that life expectancy may decrease by around 3–4 years for every decade earlier in the beginning of DM, a trend that presents a major challenge to future public health systems [7].

Despite comprehensive documentation of the effects of T1DM in individuals aged 10–24 years and T2DM in patients aged 15–39 years, a systematic analysis of the long-term trends of both diabetes types in the adolescent population remains insufficient, particularly concerning regional, gender, and socio-demographic index (SDI) levels. This study systematically analyzed the disparities in the disease burden of DM from 1990 to 2023 across various regions, genders, ages, and sociodemographic index subgroups within the adolescent population, utilizing core indicators such as disability-adjusted life years (DALYs), mortality, morbidity, and prevalence. Additionally, it conducted future disease burden predictions and assessments of health inequality to furnish a scientific foundation for disease prevention and the formulation of public health strategies for this demographic.

## Methods

### Data sources

The data for this analysis were obtained from the 2023 GBD database, encompassing prevalence, incidence, DALYs, and mortality associated with diabetes mellitus in the adolescent population. The 2023 GBD database employs an integrative meta-regression methodology to address data constraints, producing standardized burden estimates for 371 diseases and injuries across 204 nations and regions, facilitating multi-tiered (global, regional, national) comparison research. This study quantified the disease burden of diabetes mellitus in adolescents by mortality rates, prevalence, death counts, and DALYs, stratifying the burden across 204 geographical regions by age and gender.

### SDI regional classification

The SDI integrates three fundamental determinants: national per capita GDP, educational attainment of the population (aged 15 and older), and age-specific fertility rates for individuals under 25 years. The index is measured on a scale from 0 (little development) to 1 (maximum development). In this study, all nations and territories were categorized into five SDI quintiles (high, high-middle, middle, low-middle, and low) according to their 2019 reference vaff, adhering to the established GBD stratification structure.

### Estimated annual percentage changes (EAPCs)

This study generated the EAPC by employing a time series regression model to evaluate the temporal trend of age-standardized rates (ASRs). This epidemiological methodology implements temporal age-standardized rate patterns via semi-logarithmic regression modeling:


$$\begin{array}{c} \text{ln}(\text{ASR})=\alpha +\beta \times (\text{calendar year})+\epsilon \end{array}$$

In this equation, α denotes the intercept, β signifies the slope, and ε represents the stochastic error term. The EAPC was calculated using the subsequent formula:


$$\begin{array}{c} \text{EAPC }=[\text{exp}(\beta )-\text{1}]\times \text{100}\% \end{array}$$

The 95% confidence intervals (CI) represent model-derived variance estimates. Trend directionality is ascertained using interval analysis: persistent rises required EAPC and lower confidence interval limits above 0, whereas drops require EAPC and upper confidence interval limits falling below 0.

### Health inequality analysis

Following the World Health Organization’s recommendations, the slope index of inequality (SII) and the Concentration Index (CI) were employed to evaluate both absolute and relative income-related disparities among countries. SII denotes the absolute disparity in illness burden between the highest and lowest SDI categories. A positive SII signifies a greater burden on high SDI countries, whereas a negative SII denotes a heavier load on low SDI countries. The CI is employed to evaluate the comparative discrepancy in the burden of DM between nations by fitting the Lorenz concentration curve using cumulative prevalence, incidence, DALYs, mortality, and cumulative population data. The CI is a numerical integration of the area beneath the curve, spanning from −1–1. A negative CI value signifies an increased concentration of DM burden in populations living in nations with lower SDI.

### Autoregressive integrated moving average (ARIMA) model

To forecast the future burden of diabetes mellitus among adolescents from 2023 to 2040, we employed a sex-stratified and region-stratified autoregressive integrated moving average (ARIMA) framework. For each time series — defined by disease (diabetes), measure (incidence, prevalence, mortality, DALYs), region (global and five SDI regions), and sex (female, male, both) — we constructed individual ARIMA models using the following procedure.

First, age-standardized rates for adolescents aged 10–19 years from 1990 to 2023 were extracted from the GBD 2023 database. Second, for each unique combination, the optimal model order (parameters p,d,q) was automatically selected using the auto.arima() function in R (version 4.4.1) with the **corrected Akaike Information Criterion (AICc)** as the selection criterion. The function performs stepwise searches across a constrained parameter space to identify the model that minimizes the AICc. Third, the augmented Dickey-Fuller (ADF) test was applied to determine the order of differencing (d) required to achieve stationarity. For all selected models, the Ljung-Box test was used to verify that residuals exhibited no significant autocorrelation (p > 0.05), confirming adequate model specification.

Model fit was evaluated using three key statistics: **AIC**, **Bayesian Information Criterion (BIC)**, and **log-likelihood**. The forecast horizon was set to 2040 (17 years ahead, from 2023). Prediction intervals at 80% and 95% confidence levels were constructed based on the estimated forecast error variance. All model specifications (ARIMA orders) and goodness-of-fit statistics are summarized in S1 Table. This transparent, data-driven approach ensures that model selection is reproducible and that forecast uncertainty is explicitly communicated.

### Statistical analysis

Statistical analyses were executed within the R (version 4.4.1). This investigation adhered to non-participatory research protocols with no direct engagement of human subjects or community populations. Dissemination strategies were expressly designed without incorporating participatory knowledge translation initiatives involving patient advocacy groups or community stakeholders.

## Results

### Global burden of DM in adolescents, 1990–2023

**Fig 1:**
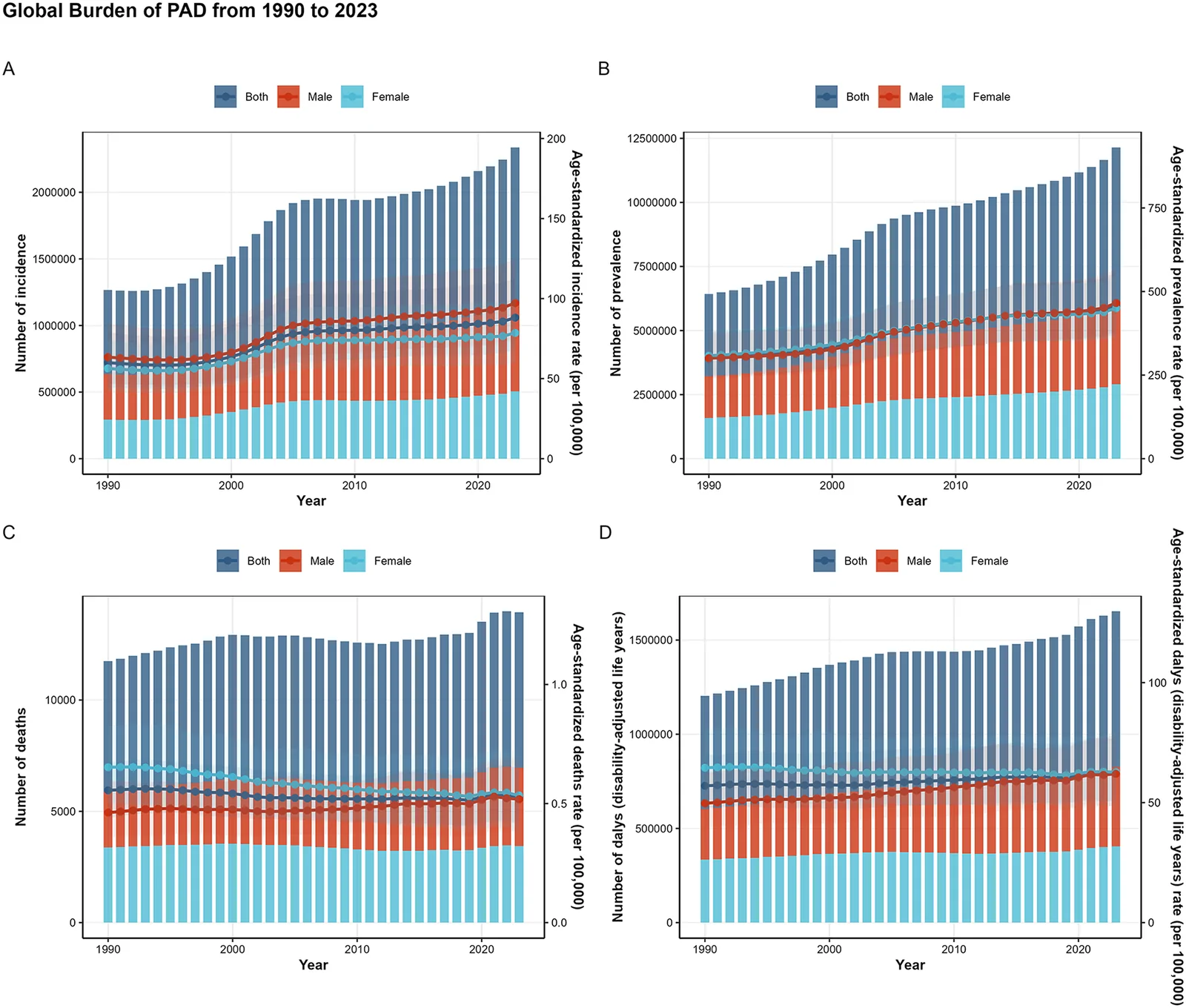
Global number and age-standardized rate of incidence (A), prevalence (B), deaths (C) and Disability-Adjusted Life Years (DALYs) (D) among adolescents from 1990 to 2023.

Between 1990 and 2023, the global prevalence of diabetes mellitus among adolescents escalated significantly. Incident cases, prevalent cases, and DALYs had significant increases during the study period, but deaths grew just somewhat. Consequently, ASIR, ASPR, and ASDR exhibited persistent rising trends, although the ASMR remained comparatively steady with a marginal decrease (Fig 1 and S1 Table).

### Prevalence of DM in adolescents

According to S2 Table, In 2023, the global prevalence of adolescent DM was estimated at 6,073,393 (95% UI: 4,928,363–7,409,672), with an ASPR of 458.07 (95% UI: 371.37–558.35). Compared with 1990 (3,215,398 [95% UI: 2,581,888–4,018,015] and ASPR 304.03 [95% UI: 244.13–379.92]), both absolute numbers and ASPR increased markedly. The global EAPC for prevalence was 1.37 (95% CI: 1.27–1.47) (S2 Table).

**Fig 2:**
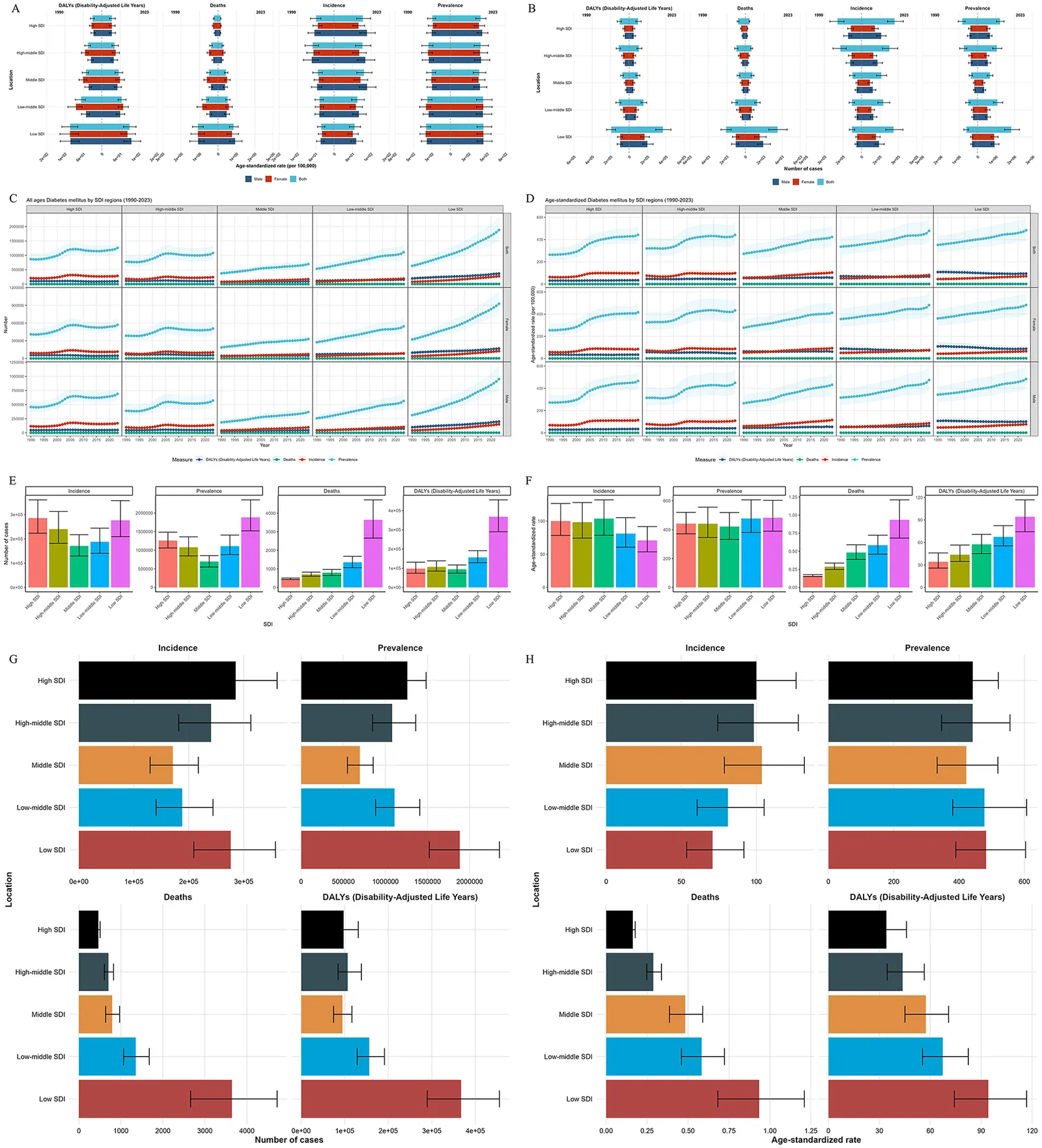
Burden of diabetes mellitus among adolescents by Socio-Demographic Index (SDI) regions. (A) Age-standardized rates of incidence, prevalence, deaths, and DALYs in 1990 and 2023 by SDI level. (B) Absolute numbers of incidence, prevalence, deaths, and DALYs in 1990 and 2023 by SDI level. (C) Temporal trends in the number of cases from 1990 to 2023 by SDI level. (D) Temporal trends in age-standardized rates from 1990 to 2023 by SDI level. (E) Comparison of the number of cases across SDI levels in 2023. (F) Comparison of age-standardized rates across SDI levels in 2023. (G) Ranking of SDI regions by number of cases in 2023. (H) Ranking of SDI regions by age-standardized rates in 2023.

Across SDI quintiles, the low-SDI group exhibited the highest ASPR in 2023 (482.25 [95% UI: 389.49–602.93]), followed by high-SDI (442.81 [95% UI: 371.87–524.00]) (Fig 2A, 2D, 2F and 2H). In terms of absolute numbers, middle- and high-middle-SDI regions contributed substantially to the global prevalence burden (Fig 2B, 2C, 2E and 2G). The EAPC for prevalence was highest in high-SDI regions (1.92 [95% CI: 1.70–2.13]) and lowest in low-SDI regions (0.94 [95% CI: 0.92–0.96]).

**Fig 3:**
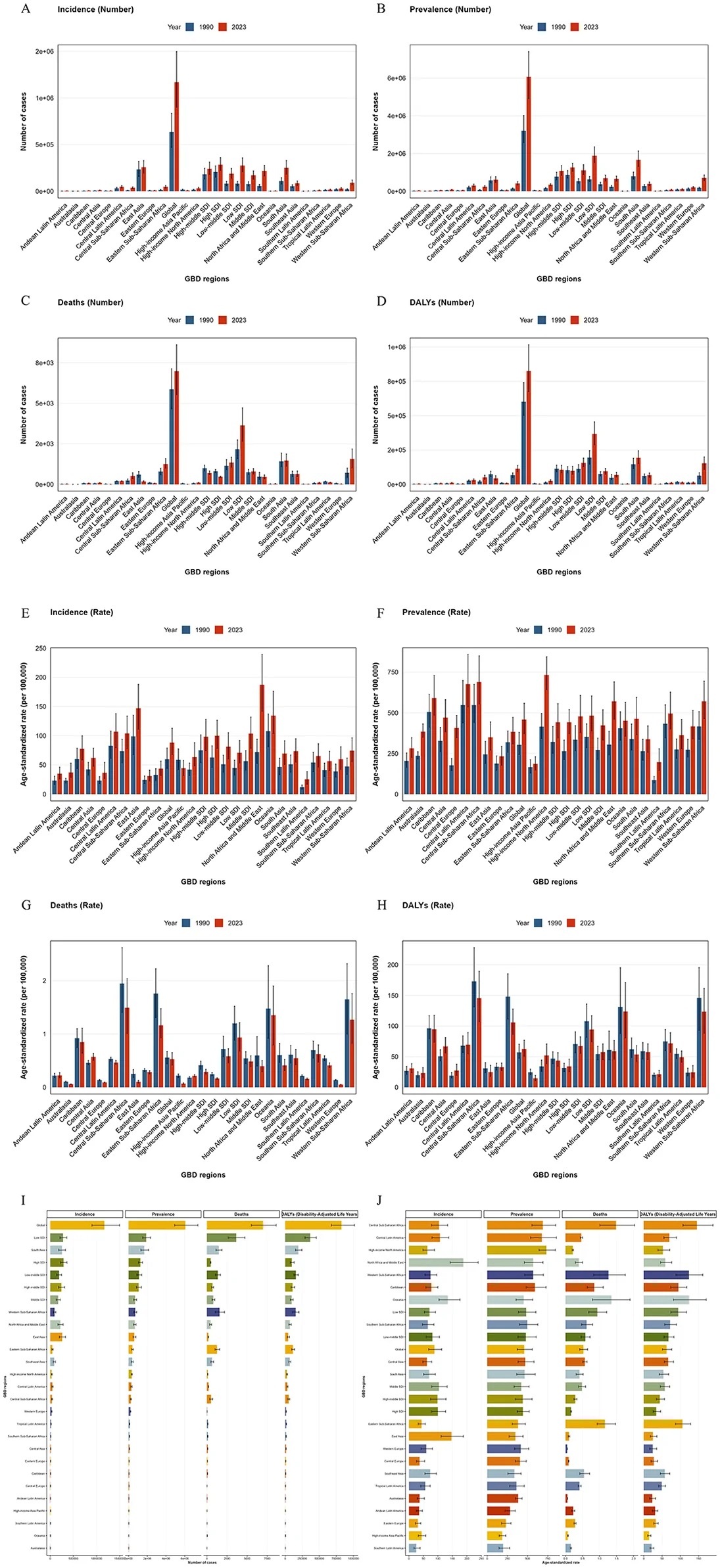
Burden of diabetes mellitus among adolescents across 21 Global Burden of Disease (GBD) regions. (A-D) Absolute numbers of incidence (A), prevalence (B), deaths (C), and DALYs (D) in 1990 and 2023 across 21 GBD regions. (E-H) Age-standardized rates of incidence (E), prevalence (F), deaths (G), and DALYs (H) in 1990 and 2023 across 21 GBD regions. (I) Ranking of regions by absolute number of cases in 2023. (J) Ranking of regions by age-standardized rates in 2023.

**Fig 4:**
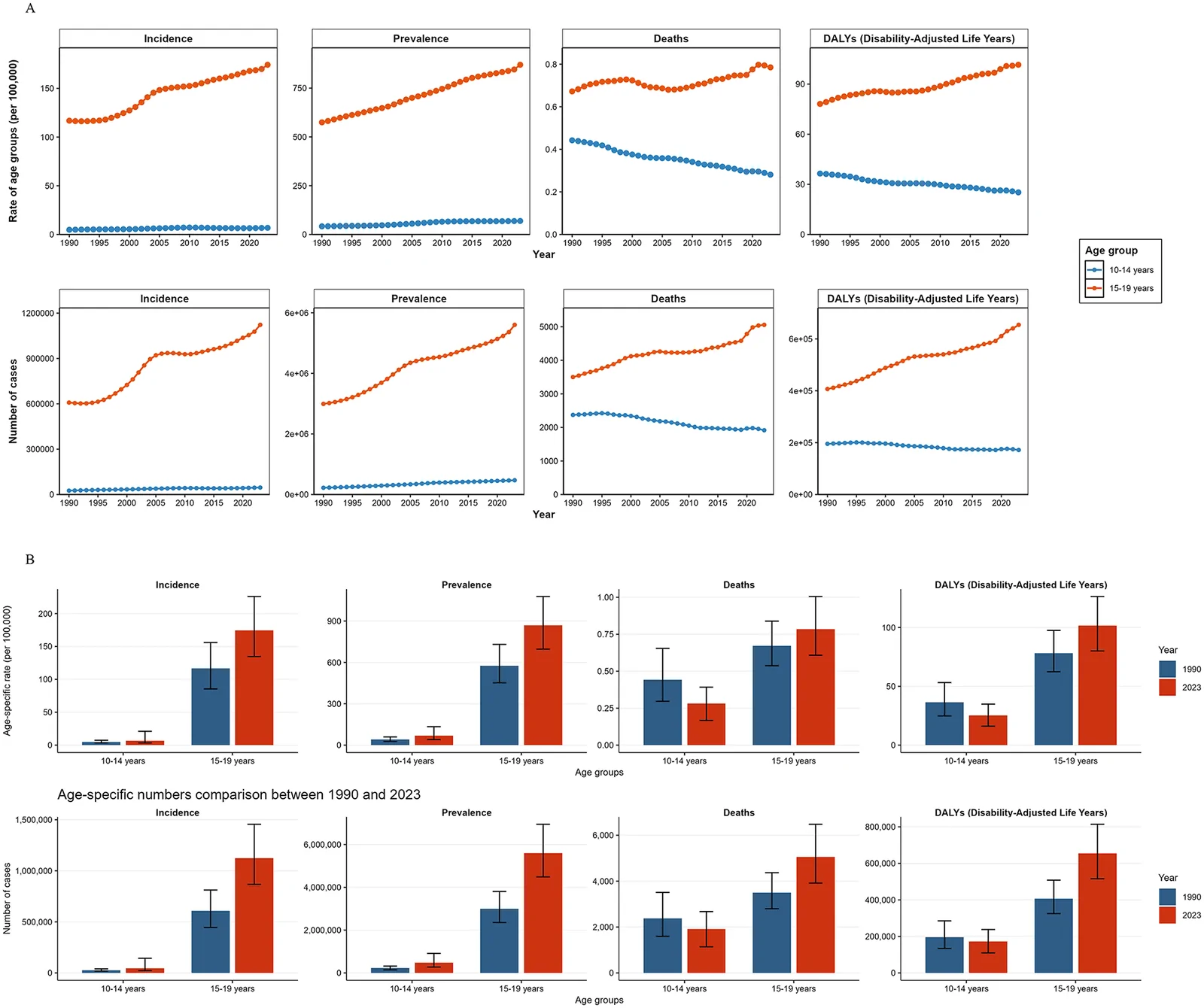
Age-specific burden of diabetes mellitus among adolescents (10-14 years and 15-19 years). (A) Temporal trends in age-standardized rates (upper panels) and absolute numbers (lower panels) of incidence, prevalence, deaths, and DALYs from 1990 to 2023 by age group. (B) Comparison of age-standardized rates (upper row) and absolute numbers (lower row) between 1990 and 2023 by age group.

**Fig 5:**
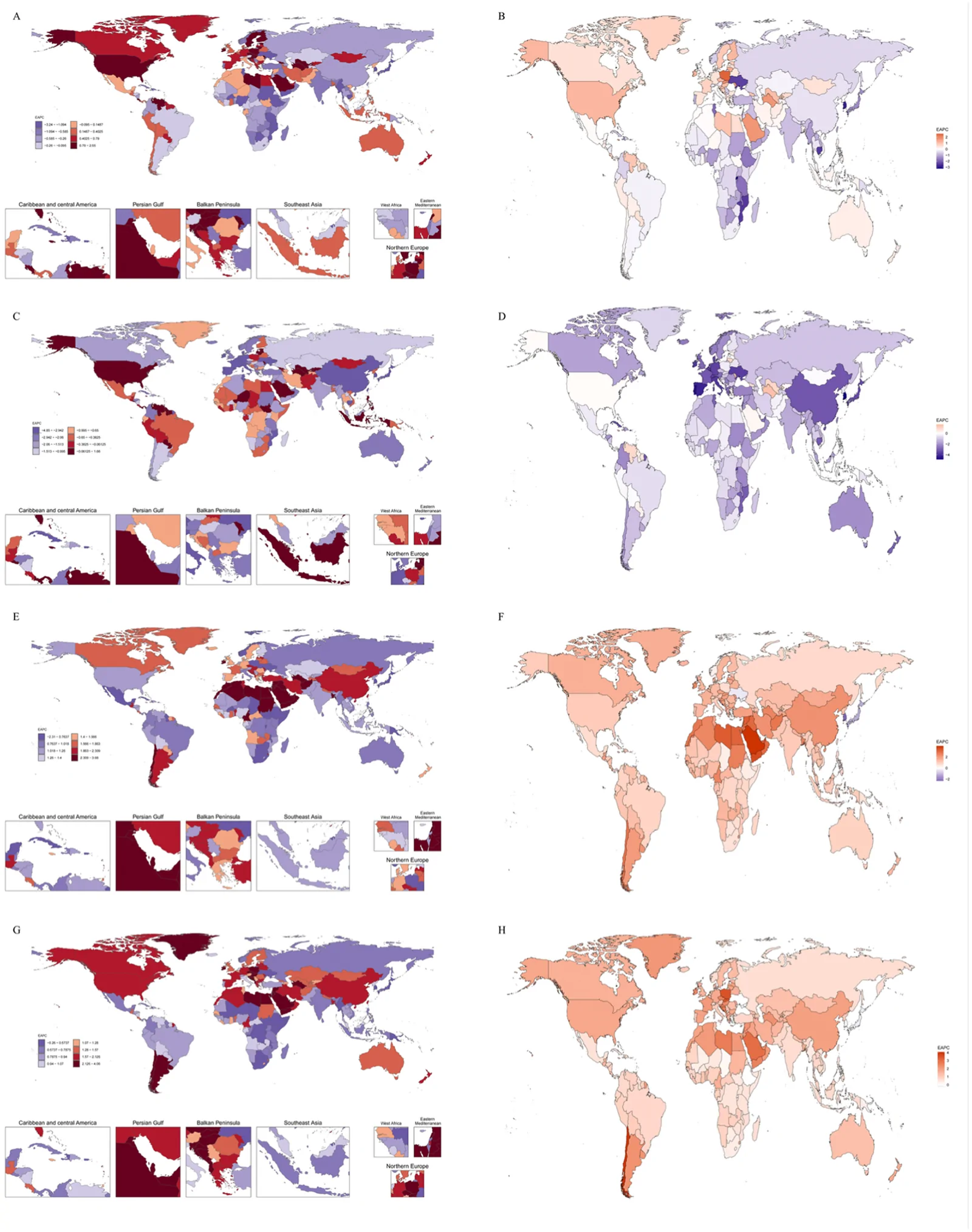
The Estimated Annual Percentage Change (EAPC) map of disability-adjusted life years (DALYs) (A, B), deaths (C, D), incidence(E, F) and prevalence(G, H).

At the regional level, High-income North America (ASPR 732.97 [95% UI: 642.65–844.15]), Central Sub-Saharan Africa (689.34 [95% UI: 555.28–849.79]), and Central Latin America (676.44 [95% UI: 536.43–858.00]) ranked among the regions with the highest ASPR in 2023 (Fig 3F and 3J). Temporal increases varied geographically, with the most rapid increases observed in Chile (EAPC 4.06 [95% CI: 3.79–4.33]), Poland (3.54 [95% CI: 3.22–3.85]), Slovakia (3.46 [95% CI: 3.19–3.73]), Kuwait (3.06 [95% CI: 2.95–3.18]), and Saudi Arabia (2.92 [95% CI: 2.61–3.23]) (S2 Table and Figs 4, 5G and 5H).

### Incidence of DM in adolescents

Globally, incident cases increased from 633,927 (95% UI: 469,794–835,132) in 1990–1,167,431 (95% UI: 900,439–1,495,161) in 2023 (S3 Table). The ASIR rose from 59.95 (95% UI: 44.43–78.98) to 87.97 (95% UI: 67.85–112.67) during the same period (S3 Table) (Fig 1A and S3 Table). The global EAPC for incidence was 1.41 (95% CI: 1.23–1.60) (S3 Table).

By SDI level, the middle-SDI regions exhibited the highest ASIR in 2023 (103.73 [95% UI: 78.69–131.99]), whereas low-SDI regions showed comparatively lower incidence rates (70.78 [95% UI: 53.53–91.60]) but increasing trends over time (EAPC 1.46 [95% CI: 1.43–1.50]) (S3 Table) (Fig 2A, 2D, 2F and 2H).

Across 21 GBD regions, North Africa and the Middle East (ASIR 187.31 [95% UI: 142.30–238.99]), East Asia (147.15 [95% UI: 111.24–187.91]), and Oceania (134.34 [95% UI: 103.96–176.37]) had the highest ASIR in 2023 (S3 Table and Fig 3E and 3J). Country-level EAPC analysis demonstrated heterogeneous temporal patterns, with several countries in the Middle East and Eastern Europe showing the fastest growth, such as Saudi Arabia (EAPC 3.85 [95% CI: 3.50–4.20]), United Arab Emirates (3.38 [95% CI: 3.09–3.68]), Kuwait (3.35 [95% CI: 3.13–3.57]), and Syria (3.27 [95% CI: 3.10–3.44]) (S3 Table) (Fig 5E and 5F).

### Deaths due to DM in adolescents

Global deaths attributable to adolescent DM increased modestly from 5,884 (95% UI: 4,746–7,175) in 1990–7,133 (95% UI: 5,624–8,830) in 2023 (S4 Table). In contrast, the ASMR declined slightly from 0.56 (95% UI: 0.44–0.67) to 0.54 (95% UI: 0.42–0.67) (S4 Table) (Fig 1C and S4 Table). The global EAPC for mortality was –0.17 (95% CI: –0.25 to –0.09) (S4 Table).

The mortality burden was disproportionately concentrated in low-SDI regions, which showed the highest ASMR in 2023 (0.95 [95% UI: 0.70–1.25]), followed by low-middle-SDI regions (0.60 [95% UI: 0.48–0.75]) (S4 Table) (Fig 2A, 2D, 2F and 2H). In high-SDI regions, ASMR declined substantially (EAPC –1.59 [95% CI: –1.83 to –1.34]) (S4 Table).

Regionally, the Caribbean (ASMR 0.85 [95% UI: 0.64–1.11]) and Sub-Saharan Africa (e.g., Central Sub-Saharan Africa 1.49 [95% UI: 1.01–2.03]; Western Sub-Saharan Africa 1.27 [95% UI: 0.83–1.76]) recorded the highest mortality rates (S4 Table and Fig 3G and 3J). EAPC maps indicated that mortality trends were heterogeneous but generally stable or declining in high-SDI regions, with notable decreases in high-income Asia Pacific (EAPC –4.19 [95% CI: –4.58 to –3.79]) and Western Europe (–3.16 [95% CI: –3.34 to –2.98]) (S4 Table) (Fig 5C and 5D).

### DALYs of DM in adolescents

Global DALYs increased from 603,211 (95% UI: 502,689–747,604) in 1990–837,874 (95% UI: 674,889–1,032,470) in 2023 (S5 Table). The ASDR rose from 57.04 (95% UI: 47.53–70.69) to 63.14 (95% UI: 50.86–77.80) (S5 Table and Fig 1D). The global EAPC for DALYs was 0.30 (95% CI: 0.26–0.34) (S5 Table).

Low-SDI regions bore the highest DALY burden in 2023, both in absolute numbers (373,735 [95% UI: 294,554–464,199]) and ASDR (95.45 [95% UI: 75.23–118.56]), followed by low-middle-SDI regions (ASDR 68.69 [95% UI: 55.26–83.89]) (S5 Table) (Fig 2A, 2D, 2F and 2H). In contrast, high-SDI regions had the lowest ASDR (34.27 [95% UI: 25.63–46.29]) (S5 Table).

At the regional level, the Caribbean (ASDR 94.79 [95% UI: 73.62–117.37]) and Eastern Sub-Saharan Africa (105.95 [95% UI: 86.61–127.75]) ranked highest in ASDR (S1 Table and Fig 3H and 3J). Country-level EAPC analysis revealed substantial heterogeneity in DALY trends, with the fastest increases observed in Poland (EAPC 2.55 [95% CI: 2.35–2.76]), Kuwait (1.73 [95% CI: 1.49–1.97]), and Turkmenistan (1.73 [95% CI: 1.31–2.14]), while notable declines occurred in the Republic of Korea (EAPC –2.86 [95% CI: –3.34 to –2.37]), Mozambique (–2.50 [95% CI: –2.77 to –2.23]), and Ukraine (–2.39 [95% CI: –2.74 to –2.05]) (S5 Table) (Fig 5A and 5B).

### Sex-specific DM in adolescents

Between 1990 and 2023, the sex differences in teenage diabetes mellitus burden evolved. In 1990, incidence and prevalence rates were elevated in males compared to females, although mortality and DALYs were marginally higher in females. Specifically, global ASIR in males was 63.38 (95% UI: 46.71–84.60) versus 56.37 (95% UI: 41.90–74.04) in females (S6 and S7 Tables); ASPR in males was 300.36 (95% UI: 240.74–377.09) versus 307.86 (95% UI: 248.70–382.90) in females (S8 and S9 Tables). Conversely, ASMR was slightly higher in females (0.65 [95% UI: 0.49–0.84]) than in males (0.46 [95% UI: 0.35–0.59]) (S10 and S11 Tables), and ASDR was also higher in females (64.68 [95% UI: 51.25–80.37]) compared to males (49.70 [95% UI: 40.05–62.78]) (S12 and S13 Tables).

By 2023, males had exceeded females in the total counts of incidence, prevalence, DALYs, and mortality. Age-standardized incidence and prevalence rates generally exceeded those of females in males. In 2023, male ASIR rose to 96.96 (95% UI: 74.82–124.59) (S6 Table), while female ASIR was 78.47 (95% UI: 59.65–99.96) (S7 Table); male ASPR rose to 465.00 (95% UI: 379.11–568.03) (S8 Table), compared to female ASPR of 450.14 (95% UI: 363.26–549.14) (S9 Table). Nonetheless, the ASMR in males (0.53 [95% UI: 0.39–0.69]) was inferior to that in females (0.55 [95% UI: 0.39–0.71]) (S10 and S11 Tables), although ASDR were analogous across the sexes: male ASDR 62.77 (95% UI: 49.04–78.67) (S12 Table) and female ASDR 63.53 (95% UI: 49.01–78.85) (S13 Table) (Fig 1). The EAPC for incidence was higher in males (1.56 [95% CI: 1.37–1.75]) than in females (1.23 [95% CI: 1.04–1.41]), while for mortality, the EAPC was positive in males (0.36 [95% CI: 0.28–0.44]) but negative in females (–0.63 [95% CI: –0.71 to –0.55]), reflecting diverging trends (S6, S7, S10 and S11 Tables).

### Age-specific DM in adolescents

Between 1990 and 2023, marked age-specific heterogeneity was observed (Fig 4). Among adolescents aged 15–19 years, incidence, prevalence, deaths, and DALYs increased steadily in both absolute numbers and age-standardized rates. In contrast, among those aged 10–14 years, incidence and prevalence showed modest increases, whereas deaths and DALYs declined over time, accompanied by decreasing ASMR and ASDR. Overall, the rising burden was primarily driven by older adolescents (15–19 years).

### Cross-national DM health inequality

**Fig 6:**
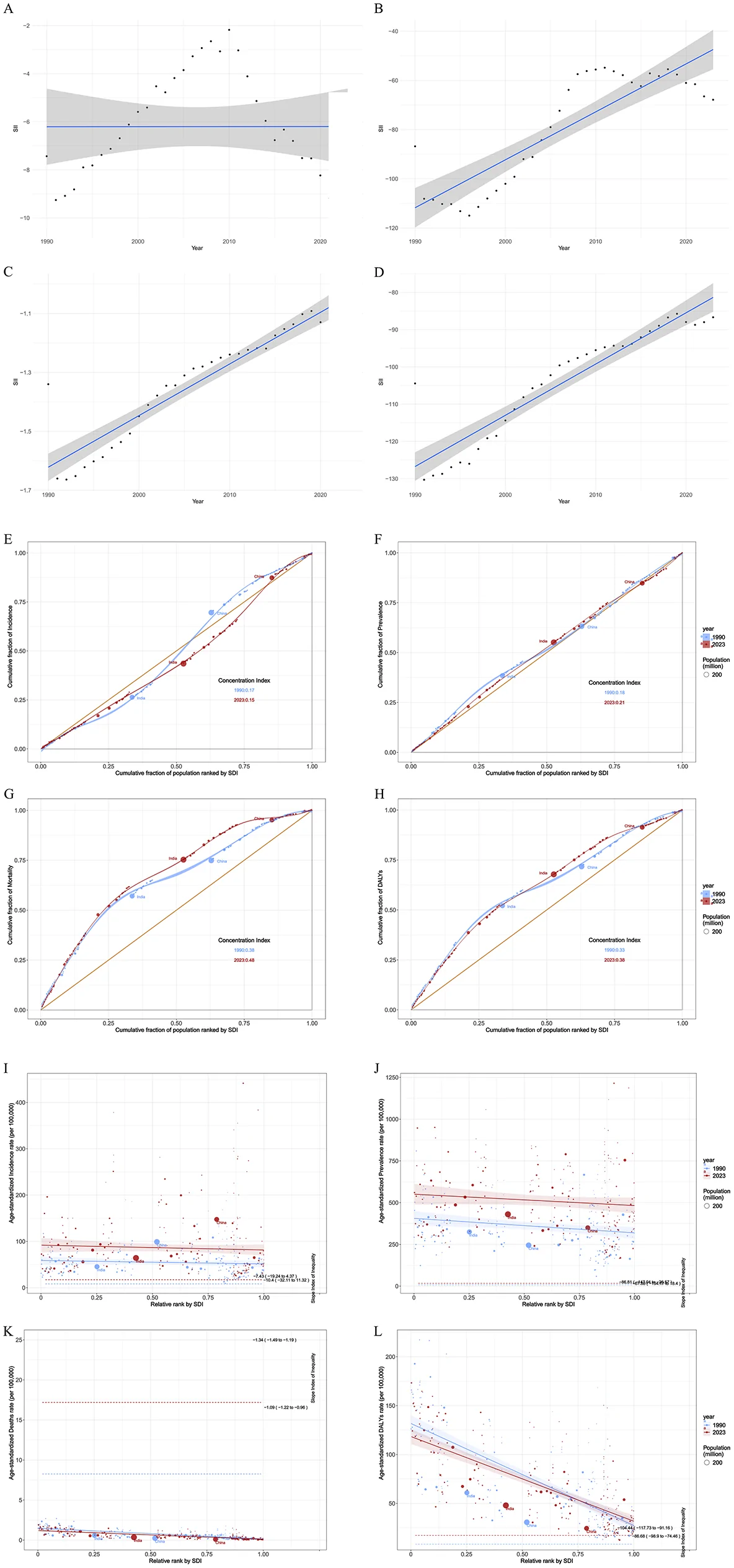
Health inequality analysis of adolescent diabetes mellitus burden. (A-D) Temporal trends in the slope index of inequality (SII) for incidence (A), prevalence (B), deaths (C), and DALYs (D) from 1990 to 2023. (E-H) Concentration curves for incidence (E), prevalence (F), deaths (G), and DALYs (H) in 1990 and 2023. (I-L) Association between age-standardized rates and relative rank by SDI for incidence (I), prevalence (J), deaths (K), and DALYs (L) in 1990 and 2023.

Between 1990 and 2023, the negative SII value for Incidence remained relatively stable, suggesting that the disparity between countries with high and low SDI persisted, albeit with a narrowing gap. Conversely, the negative SII values for prevalence, dalys, and deaths exhibited a decline, indicating a reduction in the extent of health inequality. The CI values for incidence, prevalence, DALYs, and deaths were predominantly positive and showed an upward trend, suggesting that the relative distribution of adolescent DM burden was more concentrated in higher-SDI countries when population ranking was considered. An examination of health inequality trends across different areas indicates that the overall rates of incidence, prevalence, and mortality have diminished at a sluggish rate, implying the ongoing existence of disparities between high and low SDI countries, though the gap remains relatively narrow. The pronounced slope in DALYs reveals a substantial gap between high and low SDI countries, with the health burden primarily concentrated in low SDI regions (Fig 6).

### Adolescent DM prevalence over time: 1990–2023 and forecasts to 2040

Based on the ARIMA models selected for each combination, we generated forecasts for all four indicators through 2040.

#### Incidence and prevalence.

**Fig 7:**
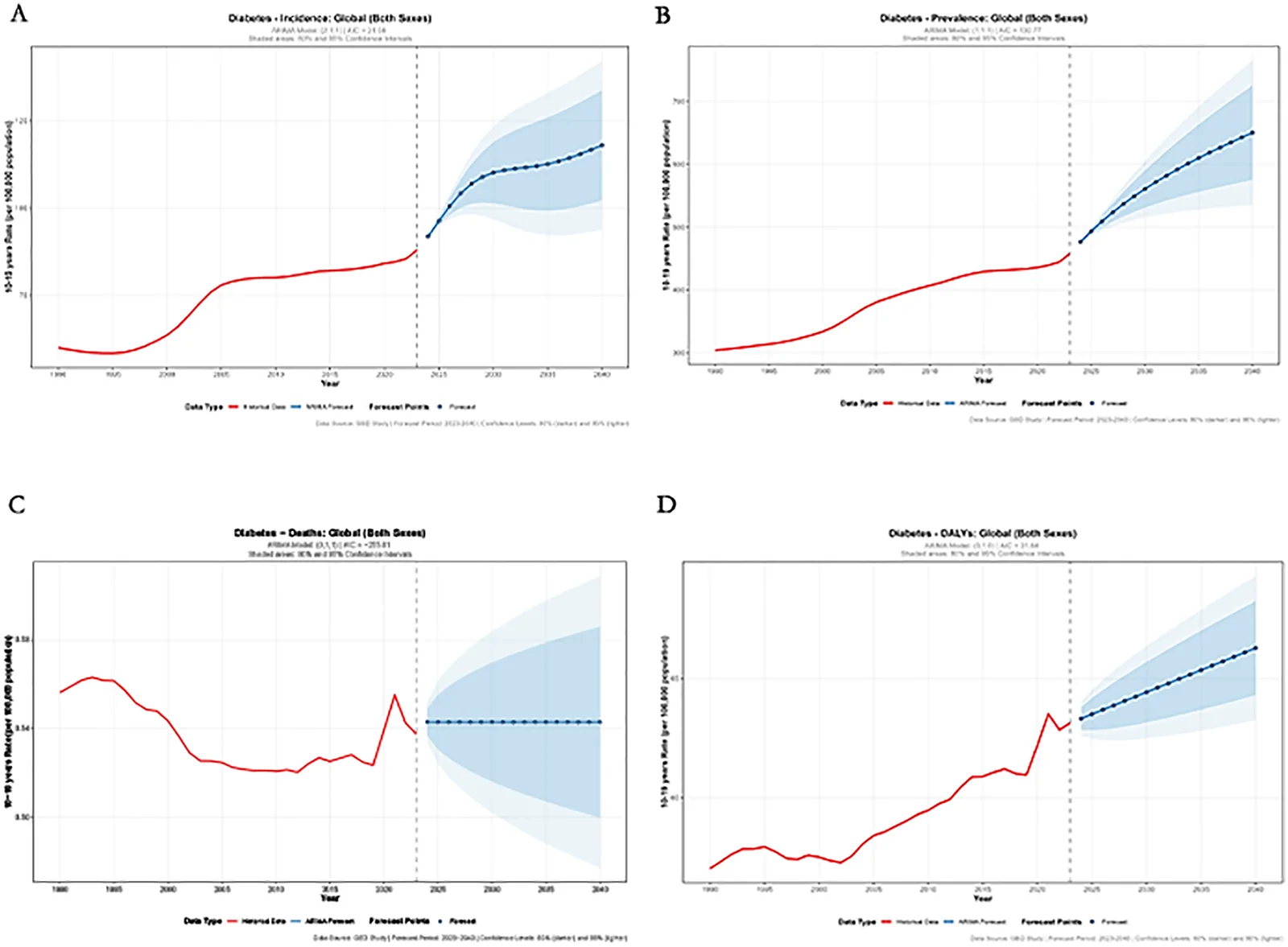
Future forecasts of diabetes mellitus (DM) global burden based on the autoregressive integrated moving average model (ARIMA) model. (A) Age-Standardized Incidence Rate. (B) Age-Standardized Prevalence Rate. (C) Age-Standardized Mortality Rate. (D) Age-Standardized Disability Rate.

The global age-standardized incidence rate (ASIR) is projected to rise from 88.14 per 100,000 (95% UI: 67.98–112.89) in 2023 to approximately 117.98 per 100,000 (95% UI: 93.81–142.15) by 2040, representing a 33.8% relative increase. Similarly, the age-standardized prevalence rate (ASPR) is forecast to increase from 458.07 per 100,000 (95% UI: 371.62–558.67) in 2023 to 650.33 per 100,000 (95% UI: 575.23–725.43) by 2040, a 42.0% relative increase. Importantly, the widening confidence intervals toward 2040 reflect increasing forecast uncertainty, which should be considered when interpreting long-term projections (Fig 7).

#### Mortality.

In contrast to non-fatal indicators, the global age-standardized mortality rate (ASMR) remained relatively stable from 1990 to 2023 (0.56 to 0.53 per 100,000). The forecast suggests a continued modest decline, reaching 0.49 per 100,000 (95% UI: 0.43–0.56) by 2040. However, the wide prediction intervals indicate that future mortality trends are less certain than those for incidence and prevalence (Fig 7C).

#### Disability-Adjusted Life Years (DALYs).

The global age-standardized DALY rate (ASDR) is projected to increase from 62.31 per 100,000 (95% UI: 50.95–76.70) in 2023 to 69.84 per 100,000 (95% UI: 63.47–77.12) by 2040 (Fig 7D), a more modest increase compared with incidence and prevalence.

#### Sex-specific forecasts.

The diverging trends between sexes observed historically are projected to persist. For incidence, the forecast ASIR for males (132.20 per 100,000; 95% UI: 104.29–160.10 in 2040) is consistently higher than for females (98.31 per 100,000; 95% UI: 81.23–115.38), with the male-female gap widening slightly from 8.31 per 100,000 in 2023 to 33.89 per 100,000 by 2040 (S1 and S2 Figs). A similar pattern is projected for prevalence: males are forecast to reach 709.13 per 100,000 (95% UI: 609.23–809.03) by 2040, compared with 608.43 per 100,000 (95% UI: 546.46–670.41) for females (S1 and S2 Figs). In contrast, sex differences in mortality and DALYs are projected to remain minimal, with overlapping confidence intervals suggesting no clear sex divergence in these outcomes (S1 and S2 Figs).

## Discussion

This study methodically evaluated the worldwide prevalence of diabetes mellitus in adolescents from 1990 to 2023. Results indicated that from 1990 to 2023, the global prevalence of diabetes mellitus among adolescents has escalated. Furthermore, this allocation of burdens exhibits a clear geographical and socio-economic gradient. High-income regions, exemplified by North America, and resource-constrained locations, such as Central Sub-Saharan Africa, represent the dual peaks of prevalence; nevertheless, the underlying causes may differ significantly [8]. In wealthy cultures, the obesity epidemic, propelled by nutritional transition—marked by the intake of ultra-processed foods and diminished physical activity—is the principal cause of insulin resistance. Conversely, in low-SDI regions, developmental origins of health and disease factors such as early-life malnutrition may influence β-cell development through epigenetic programming, laying the groundwork for long-term metabolic disorders [9,10]. The unequal nature of the burden of disease is particularly prominent in DALYs and mortality indicators. Low SDI areas carry the highest DALYs and mortality, which directly reflects their systemic shortcomings in insulin accessibility, treatment of acute complications such as ketoacidosis, and long-term complication management. For example, the high mortality rates in the Caribbean and East Africa reveal the grim reality of drug shortages and delayed first aid. The health inequality index analysis further confirms that although the prevalence gap has narrowed slightly, the significant gradient of DALYs shows that resource inequality is still increasing in improving disease prognosis. Furthermore the fastest rises in adolescent diabetes prevalence occurred in Chile, Poland, Kuwait, and Saudi Arabia, and the highest incidence in North Africa/Middle East, East Asia, and Oceania, likely driven by obesity, genetic susceptibility, urbanization, and sedentary lifestyles, highlighting the need for region-specific prevention beyond traditional high-income settings [11].

Since 2023, men have surpassed women in morbidity and prevalence. The observed sex reversal, with males surpassing females in incidence and prevalence in recent years, is consistent with growing evidence that sex and gender shape diabetes risk through both biological and social pathways. Sex differences in glucose homeostasis, adipose tissue distribution, and insulin sensitivity have been widely documented, and males tend to accumulate more visceral adiposity during adolescence, which may accelerate insulin resistance and metabolic deterioration under obesogenic environments [12–15]. In parallel, behavioral and contextual factors—such as differences in physical activity patterns, diet, and healthcare-seeking behavior—may further contribute to sex-divergent trajectories during adolescence [12,14,15]. Importantly, youth-onset T2DM has been increasing globally and disproportionately affects socioeconomically disadvantaged populations, and the relative contribution of T2DM (vs T1DM) may influence the apparent sex pattern at the population level [12,16]. Moreover, evidence from the TODAY cohort suggests clinically meaningful sex differences in metabolic phenotypes among youth with T2DM, indicating that sex may also modify disease course and downstream complications [17–20]. Nevertheless, as a population-level analysis based on modelled GBD estimates, our findings cannot determine causality, and the observed sex reversal may also reflect changes over time in screening intensity, diagnostic practices, and case ascertainment across settings. Future studies using sex-disaggregated clinical registries and longitudinal cohorts are needed to clarify the mechanisms underlying the shifting sex pattern and to inform targeted prevention strategies.

Age-specific heterogeneity suggests that the expanding adolescent DM burden is increasingly concentrated in late adolescence (15–19 years), whereas severe outcomes appear to have improved in early adolescence (10–14 years). One plausible explanation is the growing contribution of youth-onset T2DM in older adolescents, in whom pubertal insulin resistance, cumulative adiposity, and lifestyle-related risk exposures may converge to increase susceptibility and worsen metabolic control [12,13,17,21]. In addition, late adolescence is accompanied by greater self-management demands and, for some patients, transition-related gaps in continuity of care, which may further exacerbate adverse outcomes [18].By contrast, declining deaths and DALYs in 10–14-year-olds despite increasing incidence/prevalence may reflect earlier recognition and improvements in standardized pediatric diabetes care and acute complication management [19].Finally, the aggressive course and early accumulation of complications in youth-onset T2DM may amplify disability and long-term health loss in the older adolescent group [22].

Our ARIMA forecasts indicate that the global age-standardized incidence, prevalence, and DALY rates of adolescent diabetes are projected to continue increasing through 2040, whereas the mortality rate is expected to remain low and stable. This diverging trajectory — rising non-fatal burden alongside stable mortality — is clinically plausible and may reflect improvements in acute complication management, insulin access, and continuous glucose monitoring, which have reduced case fatality even as underlying incidence rises [23,24]. From a policy perspective, these projections underscore the need for sustained investment in primary prevention and in health system capacity to manage the growing pool of adolescents living with diabetes [25,26].

The persistent and widening male-female gap in both incidence and prevalence forecasts suggests that adolescent males are at increasingly higher risk of developing diabetes over the coming decades. This pattern may reflect sex differences in underlying risk factor trajectories during adolescence, such as differential insulin resistance profiles in males [27].

### Strengths and limitations

This research possesses numerous advantages. It offers a thorough and uniform evaluation of teenage diabetes mellitus burden across 204 nations and territories over a span of 34 years. Through the integration of many complementing indicators (ASIR, ASPR, ASMR, and ASDR), SDI stratification, EAPC-based trend analysis, and formal inequality measures (SII and concentration index), we provide a comprehensive assessment of illness burden and equity. Moreover, projecting to 2040 yields pertinent insights for future policy formulation.

However, some limits must be recognized. Initially, GBD estimates are derived from models and are contingent upon the availability and quality of foundational data; underdiagnosis, underreporting, and inconsistent monitoring systems—especially in resource-limited environments—may affect burden estimates despite methodological refinements. Secondly, temporal variations in diagnosis criteria, screening methodologies, and healthcare accessibility may somewhat influence the reported patterns. This study, being an ecological population-level investigation, cannot determine causal linkages or directly evaluate individual-level risk variables. Ultimately, forecasts derived from ARIMA models presume the persistence of previous trends and may inadequately consider future structural transformations, including significant policy alterations or advancements in therapy.

## Conclusion

From 1990 to 2023, the global burden of adolescent diabetes mellitus increased substantially, with marked heterogeneity across regions, sexes, and age groups. In 2023, an estimated 6.07 million adolescents were living with diabetes mellitus. The highest prevalence rate was observed in low-SDI regions, followed closely by high-SDI regions, whereas low-SDI regions bore a disproportionately high burden of DALYs and mortality. Sex patterns have shifted: by 2023, males exceeded females in both incidence and prevalence, although this observed reversal may partly reflect changes in screening and diagnostic practices. The overall increase in burden was primarily driven by adolescents aged 15–19 years. Health inequality analysis indicated persistent disparities, with a relative concentration of disease burden in higher‑SDI countries when measured on the concentration index, which may reflect differences in data coverage or healthcare access. ARIMA forecasts project that age-standardized incidence, prevalence, and DALY rates will continue to rise through 2040, whereas the mortality rate is expected to remain low and stable. These findings underscore the need for age-, sex-, and region-specific public health strategies, as well as continued investment in health system capacity to address the growing non-fatal burden of adolescent diabetes mellitus.

## Supporting information

S1 Table ARIMA model specifications and goodness-of-fit statistics. (CSV)

S2 Table Global and SDI-specific prevalence of adolescent diabetes mellitus, 1990–2023. (CSV)

S3 Table Global and SDI-specific incidence of adolescent diabetes mellitus, 1990–2023. (CSV)

S4 Table Global and SDI-specific deaths due to adolescent diabetes mellitus, 1990–2023. (CSV)

S5 Table Global and SDI-specific DALYs due to adolescent diabetes mellitus, 1990–2023. (CSV)

S6 Table Male incidence of adolescent diabetes mellitus, 1990–2023. (CSV)

S7 Table Female incidence of adolescent diabetes mellitus, 1990–2023. (CSV)

S8 Table Male prevalence of adolescent diabetes mellitus, 1990–2023. (CSV)

S9 Table Female prevalence of adolescent diabetes mellitus, 1990–2023. (CSV)

S10 Table Male deaths due to adolescent diabetes mellitus, 1990–2023. (CSV)

S11 Table Female deaths due to adolescent diabetes mellitus, 1990–2023. (CSV)

S12 Table Male DALYs due to adolescent diabetes mellitus, 1990–2023. (CSV)

S13 Table Female DALYs due to adolescent diabetes mellitus, 1990–2023. (CSV)

S1 Fig Future forecasts of diabetes mellitus (DM) global Female burden based on the autoregressive integrated moving average model (ARIMA) model. (A) Age-Standardized Incidence Rate. (B) Age-Standardized Prevalence Rate. (C) Age-Standardized Mortality Rate. (D) Age-Standardized Disability Rate.
(TIF)

S2 Fig Future forecasts of diabetes mellitus (DM) global Male burden based on the autoregressive integrated moving average model (ARIMA) model. (A) Age-Standardized Incidence Rate. (B) Age-Standardized Prevalence Rate. (C) Age-Standardized Mortality Rate. (D) Age-Standardized Disability Rate.
(TIF)
